# The Effects of Opaque and Clear Pit and Fissure Sealants on Infrared Laser Fluorescence Measurements

**Published:** 2014-06

**Authors:** Z. Bahrololoomi, M. Khodabakhsh, Y. Khaksar

**Affiliations:** a Dept. of Pediatric Dentistry, Shahid Sadoughi University of Medical Sciences, Yazd, Iran.; b Dentist; c Post Graduate Student in Pediatric Dentistry, Shahid Sadoughi University of Medical Sciences, Yazd, Iran.

**Keywords:** Caries, Laser Fluorescence, Pit and Fissure Sealants

## Abstract

**Statement of the Problem: **The purpose of placing sealants is to inhibit caries by physical closure of the pits and fissures of teeth. A device named DIAGNOdent is useful in detecting occlusal caries by employing laser fluorescence (LF). However, there are contradictory results in the influence of sealants on LF measurements.

**Purpose: **The aim of this study is to investigate the effects of two different types of fissure sealants on LF measurements.

**Materials and Method: **In this *in vitro* study, 86 extracted permanent third molars were divided randomly into two groups and clear or opaque sealant was applied on the occlusal surfaces. Two examiners performed pre- and post-seal fluorescence measurements twice with one week interval by employing DIAGNOdent device. Finally, measured values were evaluated through the statistical paired t-test by means of SPSS 17 software.

**Results: **The mean value of LF measurements increased significantly due to the application of clear sealant (*p*= 0.001) while the statistical changes in this measurement was negligible after applying opaque sealant (*p*= 0.311).

**Conclusion: **Clear sealants increase the LF measured values but opaque sealants cause almost no changes. Therefore, DIAGNOdent device is not reliable for detecting caries beneath the clear sealant.

## Introduction

Preventive dentistry deals with inhibiting occlusal caries on susceptible tooth surfaces such as pits and fissures. The application of pit and fissure sealants has been reported to be effective in caries reduction [1]. In fact, sealants physically obstruct pits and fissures to prevent bacterial colonization and penetration of fermentable carbohydrates [2-3]. It should be considered that inappropriate application of sealants and subsequent micro-leakage might lead to the progression of caries [3]. Therefore, periodic recall visits should be arranged to assure their effectiveness.

Laser fluorescent (LF) has been recently introduced to detect and quantify occlusal and dental smooth surface caries, besides visual inspection and radiographic examination. It has been reported that a device called DIAGNOdent is able to detect carious lesions beneath the apparently sound enamel by employing LF [2-5]. In fact, it is suggested that bacterial metabolites (especially  porphyrins) in carious lesions, when stimulated emit fluorescence at a specific wavelength. The emitted fluorescence is then processed and displayed as an integer between 0 and 99. This reading is regarded as LF measurement [2].

The influence of sealants on LF measurements has been examined in several studies [6-12]. Accordingly, in these studies, conflicting results were obtained regarding the effect of clear or opaque sealants on LF measurements. Some researches showed that the application of opaque sealants decreased LF reading, implying that DIAGNOdent might not be appropriate during routine check-ups to detect potential caries beneath the opaque fissure sealants. However, using clear sealants resulted in a variety of outcomes on LF measurements and was found to be lower [7, 12], unchanged [9, 11] or even higher [10] in different studies.

Gostanian et al. evaluated the ability of LF in detecting caries beneath the filled and unfilled clear sealants as well as the sealants containing titanium dioxide. Clear sealants (not containing opacifying agents) attenuated LF signals, considering the fact that filled sealants decreased LF values more profoundly than unfilled sealants (*p*< 0.001). Also, it was concluded that as the concentration of titanium dioxide reaches 0.5%, the transmission of fluorescence signals was significantly decreased [6].

In an *in vitro* study by Diniz et al., the influence of Delton clear, Delton opaque and Helioseal clear pit and fissure sealants was evaluated on infrared fluorescence measurements. By using conventional LF and an LF pen device for measurements, no statistically significant changes were observed after the application of the clear sealant, while values tended to decrease when using Delton and Helioseal opaque sealants [9].

Therefore, the aim of the current *in vitro* study was to investigate the effects of clear and opaque fissure sealants on LF measurements.

## Materials and Method

The samples in this *in vitro* study were 86 extracted third permanent molars with occlusal surfaces varying from sound to having different stages of carious lesions, yielding 245 examination sites located occlusally. Teeth with restorations, proximal caries, hypoplasia or hypomineralization were excluded from the study. Selected teeth were soaked in distilled water and kept in -20 ^o^C before the experiments. Prior to the laboratory work, the teeth were defrosted for 3 hours. Cavitron device was utilized to remove calculi from teeth surfaces, followed by cleaning for 15 seconds with water and toothbrush. To define the location of DIAGNOdent tip, buccal and lingual cusp tips were demarcated and matched to each other. Afterwards, mesial, central and distal pits on occlusal surfaces were marked as measurement sites. Finally, the photographs of all teeth were taken and the measurable points of each tooth were marked again using Photoshop software.

The employed device in this study was DIAGNOdent 2190 (LF pen; Kavo, Biberach, Germany), in which, an occlusal tip was used for the measurements. Two examiners performed all the measurements twice with one week interval. Each point was tested 3 times by the examiners and the highest result was selected as the final result.

Measurements were done both before and after the sealant application. Before measuring, the device was calibrated by calibration stone and cusp tip of the teeth. The measurement process included placing the tip of device on a selected site and rotating the tip around vertical axis until the highest fluorescence reading obtained. In each measurement, DIAGNOdent recorded two results: the moment value, which indicates the amount of caries in that specific moment, and the peak value, which represents the maximum value of the caries.

In this study, two groups of sealants including Helioseal clear and opaque (IvoclarVivadent; Liechtenstein) were used. The teeth were randomly divided into two groups (43 teeth in each group) according to the table of random numbers. Helioseal clear sealant was applied on 122 sites of 13 sound and 30 carious teeth, while 123 sites of 15 non-carious and 28 carious teeth were sealed with Helioseal opaque sealants. Before the sealant application, the occlusal surface of each tooth was etched for 30 seconds with 37% phosphoric acid gel (IvoclarVivadent; Liechtenstein). Then, the teeth were rinsed with air-water syringe for 15 seconds. Finally, the occlusal surfaces of the teeth were dried by air spray and then sealants were applied on prepared surfaces. Subsequently, sealants were cured for 40 seconds by a light-curing device (Litex 680A; 400 mw/cm^2^, USA). After curing, DIAGNOdent device was utilized again for measurements.

In addition, the defined thicknesses of 0.5 and 1 millimeter (mm) in each sealant were cured for 40 seconds on a glass slab to determine the amount of intrinsic fluorescence in clear and opaque sealants. Then, their intrinsic fluorescence was measured on a dark background using DIAGNOdent. Contents of clear and opaque Helioseal sealants are listed in Table 1.

**Table 1:** Contents of Helioseal sealants

Clear sealants	- Bis-GMA - Tri-ethylene glycol dimethacrylate (>99 wt%*) - Stabilizers and catalysts (<1 wt%)
Opaque sealants	- Bis-GMA - Tri-ethylene glycol dimethacrylate (>97 wt%) - Titanium dioxide (2 wt%) - Stabilizers and catalysts (<1 wt%)

*Percent concentration

## Statistical analysis

Paired t-test was used to compare the mean difference of fluorescence measurements before and after placing the clear or opaque sealants.

Intra-class correlation was used to assess inter- and intra-examiner reproducibility. The significant level was set at *p*< 0.05.

## Results

Table 2 compares the effect of clear and opaque sealants on LF measurements. It is obvious that although LF measurements tended to increase after sealing with both clear and opaque sealants, it was statistically significant only in clear sealant group.

**Table 2:** Comparison of the mean value of pre and post- seal fluorescence measurements regarding the types of sealants

**Type of sealants**	**LF before sealing** **Mean ± SD**	**LF after sealing** **Mean ± SD**	**P value**
Clear	22.95 ± 27.22	30.21 ± 30.52	0.001
Opaque	18.72 ± 25.04	21 ± 28.92	0.311

The inter-class correlation ranged from 0.983 to 0.994 for intra-examiner reproducibility and from 0.982 to 0.986 for inter-examiner reproducibility. The fluorescence values of sealant samples with 0.5 and 1.0 mm thicknesses are shown in Table 3.

**Table 3:** Intrinsic fluorescence values of sealant samples with thicknesses of 0.5 and 1.0 mm

	**0.5 mm**	**1 mm**
Opaque Helioseal	2	5
Clear Helioseal	2	2

This reproducibility indicates the similarity between the results of measurements carried out with sealants with different thicknesses. 

## Discussion

Pit and fissure sealant therapy is considered as an effective procedure to prevent occlusal caries. However, periodic assessment of the sealed surfaces should be performed in order to avoid underestimation of caries beneath the applied sealants. A possible method to achieve this goal is to employ DIAGNOdent LF [4, 7, 11, 13].

When the extracted teeth are stored in -20^o^C, LF readings are not affected [14]. Due to this fact, teeth were kept in the aforementioned temperature prior to experiments.

Anttonen et al. concluded that teeth surfaces with visible plaque should be cleaned by rotating instruments and water spray prior to LF readings [15]. Also, Hosoya et al. have shown that tooth polishing paste might interfere with LF readings [16]. Therefore, in this study we preferred not to use pastes and the teeth were cleaned with water and toothbrush.

As clear sealants allow visual examination of carious lesions beneath the sound enamel, they seem to be the best option to clinically detect the caries [9]. Therefore, caries detection beneath the opaque sealants seems more essential.

In our study, DIAGNOdent LF readings were significantly increased after sealing the occlusal surfaces with clear Helioseal, which might result in potential caries overestimation in sealed occlusal surfaces.

As it was mentioned, different studies have found a variety of outcomes in LF measurements after the application of sealants. The differences among studies might be attributed to the differences in methods, such as type of teeth, sample size and storage medium, disease level at the examination sites, etching time and the materials that have been used [10].

A significant increase in LF readings after clear sealant application has been also reported in Asksroglou et al. study, which was done on primary molars [10].

Diniz et al. and Krause et al. concluded that the effect of clear sealants on LF measurements is not statistically significant [9, 11]. Therefore, they proposed that employing DIAGNOdent device was a suitable supplementary method for caries detection beneath the clear sealants. In studies conducted by Diniz et al. and Krause et al., Delton sealant was used, while we performed our study on Helioseal sealants.

Deery et al. and Sonmenz et al. have concluded that fluorescence measurements was significantly decreased after applying clear sealants [7, 12].

The different media in which the teeth were stored prior to experiments, can lead to different results. In the study of Deery et al., storing media was 1% aqueous thymol solution and in the study by Sonmenz et al., teeth were kept in distilled water, while in our study teeth were frozen at -20 ^o^C before the experiment.

In our study, DIAGNOdent measurements showed no significant difference after sealing occlusal surfaces with opaque Helioseal sealant. It indicates that it is possible to detect caries beneath the opaque sealants by means of LF. In studies by Diniz et al. and Rodrigues et al., LF reading decreased significantly after sealing with Delton and Helioseal opaque sealants [9, 17]. Since etching affects the scattering and translucency of enamel [9], etching time should also be considered. Etching time in Diniz et al. and Rodrigues et al. studies was 60 seconds while in our study, surface etching was performed for 30 seconds.

Also in the study by Asksroglou et al., LF measurement significantly decreased after applying opaque sealants [10]. This study, however, was done on primary molars and Delton opaque sealant was applied on occlusal surfaces.

Krause et al. who also investigated the effects of composite fissure sealants on infrared LF measurements showed that LF values significantly decreased after sealing with the opaque sealants [11].

The most common opacifying filler in sealants is TiO_2_. This filler facilitates the application of sealant and its visual assessment in recall visits [6]. As the TiO_2_ content increases, fluorescence transmission is attenuated. This indicates that the existing TiO_2_ in sealants might interfere with the fluorescence transmitted from caries or laser devices. Delton opaque sealants contain 1.5% TiO_2_, and also SiO_2 _[10]. Helioseal opaque sealant, which was used in our study, contained 2% TiO_2_.

The existing fillers and TiO_2_ in sealants create intrinsic fluorescence and the scattering of laser beam. Consequently, they can cause false positive and false negative results. Furthermore, other components of different sealants might affect LF readings.

It has been shown that the intrinsic fluorescence of clear and opaque sealants significantly affects LF readings, as this intrinsic fluorescence is not discriminable from that of carious lesions [6]. Therefore, it might lead to increased false positive results and therefore caries overestimation. In this study, the intrinsic fluorescence of both clear and opaque sealants in 0.5 and 1 mm thicknesses was determined. The intrinsic fluorescence in clear sealant was the same in both thicknesses; however, in opaque sealants it was increased three units in the 1 mm thickness. As in this study, the amounts of caries were not standardized and the sample of study also consisted of cavitated teeth, which need thicker sealant, this might account for the insignificant changes of LF readings in opaque sealants.

## Conclusion

In conclusion, clear sealants may increase the measured LF values, which can lead to the overestimation of caries beneath them. While opaque sealants may cause almost no changes, DIAGNOdent device seems valuable for detection of hidden occlusal caries beneath the opaque sealants. Further studies should be performed with different types of sealants as clinical trials to address the influence of pit and fissure sealants on LF measurements more precisely. 
